# Validation of exposure assessment and assessment of recruitment methods for a prospective cohort study of mobile phone users (COSMOS) in Finland: a pilot study

**DOI:** 10.1186/1476-069X-10-14

**Published:** 2011-03-08

**Authors:** Sirpa Heinävaara, Kari Tokola, Päivi Kurttio, Anssi Auvinen

**Affiliations:** 1 Radiation and Nuclear Safety Authority, Research and Environmental Surveillance, Health Risks and Radon Safety, Laippatie 4, 00880 Helsinki, Finland; 2 Tampere School of Public Health, 33014 University of Tampere, Finland

## Abstract

**Background:**

The aim of the study was to evaluate the agreement between self-reported and operator-derived estimates of call time based on a three-month monitoring period, as well as the consistency of mobile phone use over time. Alternative approaches to improve participation in a cohort study of mobile phone users were also compared.

**Methods:**

A total of 5,400 subjects were identified from network operators' subscriber databases for recruitment to the pilot study. Operator and questionnaire data were used to quantify mobile phone use. Operator data were available for a subset of the subjects for a three-month period in three consecutive years. We also evaluated the effect of the length of the questionnaire and one- or two-phase recruitment on participation.

**Results:**

The average response rate for both questionnaires and recruitment procedures was 12%. The response rate was not affected by the length of the questionnaire or the recruitment method.

Operator data were available for 83% of the participants for 2007, the first study year. The agreement between self-reported and operator-derived call times decreased with the level of use among intermediate and heavy mobile phone users. During 2007-2009, mobile phone use increased fairly constantly over time.

**Conclusions:**

The agreement between self-reported mobile phone use and operator databases was moderate and overestimation of the call time by participants was common. A prospective cohort study would be feasible in Finland, although the potentially low participation rate would increase the resources required for recruitment.

## Background

Earlier studies on the health of mobile phone users have mostly been case-control studies of brain tumors [1-3]. Indicators used for radiofrequency (RF) electromagnetic field exposure from mobile phone use have included the number of calls made and received, duration of calls, laterality of use and duration of mobile phone use (in years). In most studies, exposure assessment has been based on self-reported use with only a few exceptions utilizing network operator records [4-6]. Retrospective assessment of exposure based on self-report is problematic, since the accuracy of such estimates is poor and prone to recall bias [7-10]. A further source of error is selection bias shown in some studies to result from the low participation rate of controls who do not use mobile phones [11,12]. These systematic errors seem difficult to avoid entirely in case-control studies. Furthermore, the pooling of results from several studies in meta-analyses does not remediate the problem of bias. Therefore, strategies to avoid such distortions should be sought for future studies, and a prospective cohort design is one such approach. A European collaborative cohort study, known by the acronym COSMOS, has already been initiated in Denmark, Sweden, Finland and the UK, and it will be launched in the Netherlands [13].

Although most studies have focused on the cancer risk, RF exposure from mobile phone use may cause not only brain tumors but possibly also other diseases and symptoms [14]. As mobile phones are used by more than 90% of the adult population in many industrialized countries, even a minor excess risk would have a major public health impact.

Due to both methodological advantages and the wider scope of the outcomes, the need for a large, prospective cohort study on the health of mobile phone users has been recognized in several evaluations [15-17]. Unlike case-control studies, a cohort study can include several outcomes, for instance neurological and cerebrovascular diseases in addition to intracranial tumors. The selection of relevant end-points is challenging, as no biological mechanism has been identified for RF fields [17]. However, as the RF field is highly localized, it seems logical to evaluate the potential health effects on organs and tissues in proximity to the actively transmitting phone during calls. As the mobile phone is mostly held on the ear (with the exception of use with hands-free devices or on speaker mode), RF exposure is largely limited to the head and neck region. The vast majority of previous epidemiological studies on the health effects of mobile phone use have focused on intracranial tumors, with a few studies on salivary gland tumors and lymphomas. The rationale has been an analogy to ionizing radiation, with the major health effect of increased cancer risk. However, as RF fields do not have sufficient energy to break chemical bonds or induce mutation with a similar mechanism to ionizing radiation, other potential health effects are also plausible. Furthermore, several cross-sectional studies have revealed a higher frequency of various symptoms affecting well-being (including sleep disturbances and headaches) among people exposed to RF [18-24]. Such effects may be due either to psychological distress because of the perceived risk (nocebo) or neurophysiologic effects, which can also be studied in a cohort study.

Uncertainty in exposure assessment due to the retrospective assessment inherent for case-control studies has been a key limitation of previous investigations. A handful of studies have demonstrated only moderate consistency between self-reported mobile phone use and recorded estimates (obtained from operator databases and software modified phones), with overestimation by a factor of up to two or more [7-10,25,26]. However, validation studies are likely to have overestimated the precision of interviews, as they have addressed periods of mobile phone use covering some months, while the recall period in epidemiologic studies on the health effects of mobile phone use has spanned several years. In a cohort study, the limitation due to recall bias (exposure information affected by disease status) and random error due to retrospective reporting can be effectively overcome. In a prospective study, exposure assessment can utilise objective data from operator databases on the number and duration of calls (in the COSMOS study this has been proposed as annual downloads covering a three-month period). Exposure assessment can be supplemented by questionnaire data on the mode of use (such as hands-free devices, side of head) and circumstances of use (urban-rural, stationary-moving and indoor-outdoor). The annually updated operator data can be used to define the exposure time and the questionnaire results are informative with respect to the intensity and location of exposure (as determinants of the output power and hence field strength).

To increase cost and information effectiveness, a cohort study should cover a wide range of exposures in order to provide an exposure contrast. Stratified sampling of the customers in operator databases, with stratification according age, sex and the level of mobile phone use, was considered optimal for this purpose [13]. Information on mobile use without contacting the subjects themselves is only available through mobile phone operators. A challenge is obtaining all the relevant information from study subjects with multiple phones, particularly those owned by the employer.

We assessed the agreement between the self-reported use of mobile phones and operator data, as well as the consistency of use during subsequent time periods (three months in a three-year period) within a pilot study investigating the feasibility of a cohort study. The pilot study was also used to evaluate whether the response rate was affected by the length of the questionnaire or the recruitment procedure (consent form and questionnaire sent separately or together). Furthermore, we tested the collaboration with network operators in both the recruitment of study participants and exposure assessment.

## Methods

We were able to establish collaboration with all three major Finnish mobile network operators. Two of them allowed recruitment of their customers and agreed to provide mobile phone data on those who participated in the study. These two network operators selected random samples of eligible subjects out of subgroups of their customers in autumn 2006 using pre-defined criteria, including only private subscribers with a service contract for at least six months who had not denied the transfer of their information to a third party. Subgroups of subjects were formed according to the monthly call time (light, intermediate and heavy users), age (18-29, 30-39, 40-49, 50-59, 60-69 years) and sex. All strata, i.e. combinations of the subgroups, were of the same size. Monthly call time was based both on incoming and outgoing calls, and was calculated as the mean of the monthly average calling time over the previous six months. Light users had less than 30 minutes of call times per month, intermediate users 30-359 minutes per month, and heavy users at least 360 minutes per month. The selection of cut-points was based on a previous study [9]. Age and sex were defined based on the unique personal identification number of the subscription owner. Network operators provided the contact information for eligible subjects to an external service provider who sent the invitations to participate in the pilot study. The external service provider was used because the operators' information on the monthly call time could not be released to the researchers without the subject's consent.

**Figure 1:**
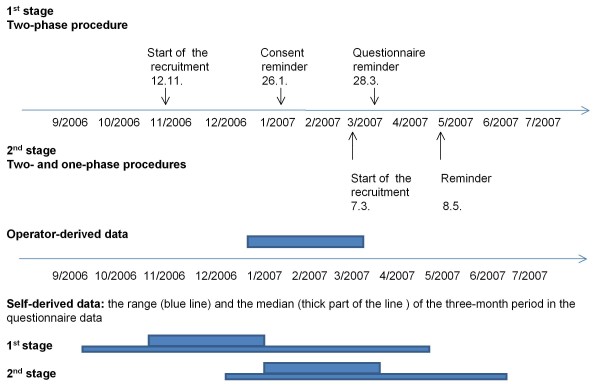
**Time lines of study recruitment and the periods of operator-derived and self-reported data**.

We compared two approaches for mailing the study material. In the first stage (December 2006), the service provider mailed an invitation letter, an informed consent form and a return envelope (postage paid) to 3,000 persons. Subjects were sent the study questionnaire in a separate mailing after they had signed and returned the consent form (two-phase procedure). In the second stage (in March 2007), a new sample of 2,400 subjects was identified from one operator's customer database. Half of them received the questionnaire in the first mailing (together with the information leaflet and consent form, one-phase approach) and the other half only after returning the consent form (two-phase approach, as in the first stage). The timelines of the study are presented in the upper half of Figure 1.

In both mailings, one network operator added a letter explaining its role in the study to the customers, whereas the other one did not. Non-responders were sent a reminder 9-10 weeks after the mailings. In the first stage, a press release was issued at the time of the first mailing.

We assessed the effect of the questionnaire length on participation. In both mailings, two versions of the questionnaire were used (long and short), with one-third of the subjects receiving the shorter questionnaire. A total of 43 core questions over 14 pages were included in both long and short questionnaires pertaining to mobile phone use, well-being and health, symptoms, and risk factors for the diseases defined as outcomes. The questions on mobile phone use covered past mobile phone use (weekly number and duration of calls made and received using analogue and digital phones), current mobile phone numbers and network operators, and self-reported mobile phone use (daily number and duration of calls made and received during the previous three months). The long questionnaire included an additional six pages with 36 questions.

Both network operators provided mobile phone use data for study participants for the first year, in early 2007. To assess the variability in mobile phone use over time, additional data were obtained for 2008 and 2009 from one of the operators. The reported mobile phone numbers linked the records to the study participants. The operator data comprised the monthly number of incoming and outgoing calls, and the starting time and duration of individual calls over the three-month period.

### Statistical methods

The association between the self-reported and operator-derived data was examined with Spearman's rank correlation and with the Bland-Altman method. The data were analyzed using Stata statistical software (version 10, Stata Corp., College Station, TX).

The study protocol was reviewed by the ethical committee of the Pirkanmaa Hospital District (reference number R04179).

## Results

### Recruitment

**Figure 2:**
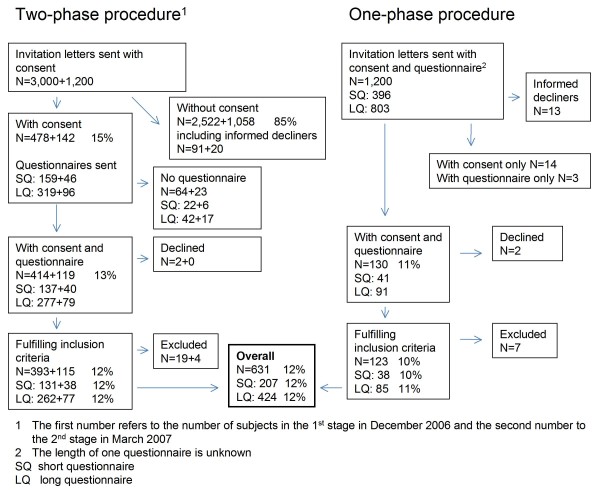
**The recruitment of the study participants using the two- and one-phase procedures with short and long questionnaires**.

The recruitment of the study participants using the two- and one-phase procedures with short and long questionnaires is illustrated in Figure 2. Approximately 17% (891/5,400) of the study subjects responded to the invitation to join the study. A total of 124 persons (2% of the study subjects and 14% of the respondents) informed the study personnel that they refuse to participate in the study (informed decliners). The most commonly cited reasons included old age or a lack of interest. Subjects younger than 18 years or older than 69 years (N = 11), those who did not belong to the operators' samples (N = 11), or those who did not provide a mobile phone number on the informed consent form (N = 8) were excluded.

The overall participation rate for both questionnaires and recruitment methods was 12% (631/5,400, Figure 2). The subjects who received the long questionnaire participated equally frequently to those with the short version (12% vs. 12%). The participation rate was slightly higher in the first than the second stage (13%, 393/3,000 and 10%, 238/2,400). In the second stage, there was no difference in the success of recruitment between the one- and two-phase procedures (10%, 115/1,200 vs. 10%, 123/1,200). In the first stage, the reminder after the invitation letter resulted in additional consent forms being returned (46%, 221/478), but relatively few further questionnaires were returned after the questionnaire reminders (7%, 31/414). The letter from the operator did not increase the response rate (12% with letter vs. 17% without letter in the first stage).

The overall participation rate was highest among the oldest people receiving the invitation (15%) and lowest (9%) in the age group of 40-49 years (Table 1). Participation was slightly more common among women than men (13% vs. 11%) and increased with the level of mobile phone use. The response rate was thus highest among the oldest heavy users (21%), and lowest among the middle-aged (40-59 years) light users (8%).

**Table 1:** Respondents and response rates (%) by age group, sex and mobile phone use, and by stage.

	**1**^**st **^**stage**	**2**^**nd **^**stage**	All
**Age group (years)**	N (%)	N (%)	N (%)

18-29	80 (13)	40 (8)	120 (11)

30-39	75 (13)	59 (12)	134 (12)

40-49	58 (10)	40 (8)	98 (9)

50-59	76 (13)	37 (8)	113 (10)

60-69	104 (17)	62 (13)	166 (15)

**Sex**			

Male	179 (12)	102 (9)	281 (11)

Female	214 (14)	136 (11)	350 (13)

**Call time (minutes per month)**			

0-29	99 (10)	62 (8)	161 (9)

30-359	138 (14)	76 (10)	214 (12)

360-	156 (16)	100 (13)	256 (14)

Usually, the mobile phone subscriber was also the user of the subscription, since the age group defined by the network operators and that obtained from the consent form was the same for 96% (717/747) of study participants, and the sex was the same for all.

### Validation

The lower half of Figure 1 shows the three-month periods for which the operator-derived and self-reported mobile phone use data were gathered. The period of self-reported data ranged widely. The best match between the operator records and self-reported data was obtained with the 2^nd ^stage procedure.

For the first year (2007), mobile phone data were received for January-March from the operators for 83% (526/631) of the study participants. The comparison of the operator-derived and self-reported mobile phone use was limited to users with one mobile phone subscription alone (79%, 418/526). The mean monthly duration of incoming and outgoing calls was 314 minutes with an average of 80 calls (median 215 minutes and 62 calls).

In the sampling phase, the operator whose customers were recruited in both stages defined the sub-groups according to the level of use based on a six-month period from May to October 2006. For the other operator whose customers were included in the 1^st ^stage only, the six-month period from March to August 2006 was used. Mobile phone use differed between this six-month period preceding the start of the study and the first monitoring period (January-March 2007): More than one-third (156/418) of the subjects changed their level of phone use enough to switch to another category, often to a category with a longer call time (102/418).

**Figure 3:**
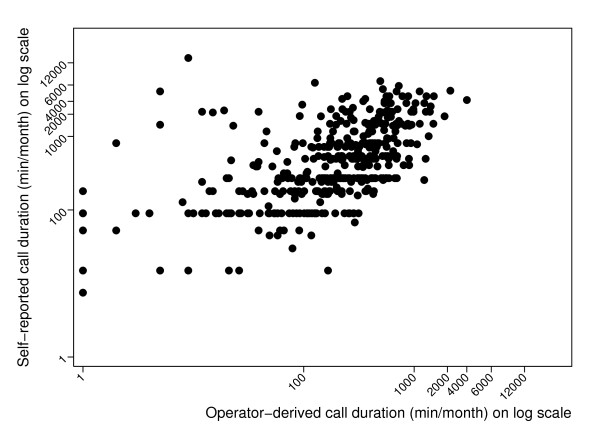
**The operator-derived and self-reported monthly call duration (minutes per month) on log scale among users with one mobile phone alone (Spearman's rank correlation coefficient rho = 0.60 with 95%CI [0.54, 0.66])**.

**Figure 4:**
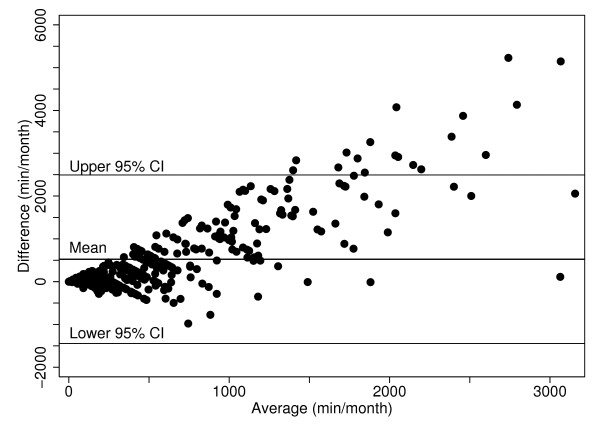
**The Bland-Altman plot for the association between the operator-derived and self-reported monthly call durations (minutes per month)**.

The self-reported averages of monthly call times were moderately correlated with the operator-derived data (Spearman's rank correlation coefficient rho = 0.60 with 95%CI [0.54, 0.66], Figure 3). The association between the self-reported and operator-derived monthly averages for the call duration is also illustrated with the Bland Altman plot (Figure 4), which clearly indicates an error in self-reported estimates increasing in proportion to the call time. One subject with an exceptionally high self-reported average and low operator-derived average was excluded. The mean difference between the self-reported and operator-derived monthly averages of call duration was 523 minutes with 95%CI [-1,446, 2,493]. The median ratio between the self-reported and operator-derived mobile phone use was 2.2 for the call duration and 1.6 for the number of calls. The sensitivity of the results was assessed by restricting the analysis to the study subjects for whom the time period of self-reported and operator-derived data differed by one month or less. For these 118 study subjects, the Bland-Altman plot was very similar to Figure 4, with the mean difference between the self-reported and operator-derived monthly averages of call duration being 476 minutes and 95%CI [-1,212, 2,164].

After the initial recruitment phase, mobile phone data were received from one operator alone for 423, 386 and 398 study subjects in 2007, 2008 and 2009, respectively. The monthly call time and the number of calls were both strongly correlated between the consecutive years (Spearman's rank correlation coefficients, rho ~ 0.8 for call duration and rho ~ 0.88 for the number of calls). Operator-derived data received for all three years for the same study subjects are presented in Table 2.

**Table 2:** Descriptive statistics of operator-derived mobile phone data that were received for 346 study subjects for all three years 2007-2009.

Year	2007	2008	2009
**Call time (minutes per month)**			

mean	298	316	342

median	186	220	221

**Number of calls (per month)**			

mean	70	77	80

median	52	54	57

## Discussion

Our findings indicate overestimation of self-reported mobile phone use in comparison with traffic data obtained from network operators. This implies that repeated data downloads from operator databases are required for valid exposure assessment in a prospective cohort study. A second key finding was that the participation rate was unaffected by the length of the questionnaire or the recruitment method.

Self-reported retrospective exposure assessment is subject to substantial error, both systematic and random. Risk estimates can be severely affected by random error in exposure estimates [27]. Non-differential random errors typically deflate risk estimates for dichotomous and continuous exposures (and trend effects for ordered polytomous exposures) towards the null (no effect), reduce their precision, and may therefore mask true effects. Such errors rarely result in spurious associations in the absence of true effects. However, when a categorical (polytomous) exposure indicator is used, random error can result in either under- or overestimation of the effect (downward or upward bias). One possibility to correct for the low precision is regression calibration, which has the potential to improve the control of random error and yield a more realistic dose response. Its anticipated impact on the outcome in an analysis is a steeper exposure-effect gradient, given a true effect. Likewise, systematic error may bias the risk estimates in either direction. Differential error can also bias the results in any direction, depending on the differences between cases and controls in case-control studies and exposure groups in cohort studies.

Our results are in agreement with earlier studies assessing the reliability of self-reported mobile phone use. In the UK [8], a reasonably good correlation was reported between telephone companies and the self-reported mobile phone use among 90 study subjects over a six-month period. The agreement was slightly better for the call duration (kappa = 0.50, r = 0.60) than the number of calls (kappa = 0.39, r = 0.48), but overestimation was substantial for both of these measures (overestimation by factors of 2.8 and 1.7, respectively). In a study conducted in the US [25], a reasonably good correlation was reported in call duration between telephone company records and self-reported mobile phone use (r = 0.74). In Germany [26], a weak correlation (r = 0.34) was found for the average call duration and a moderate correlation for the number of calls (r = 0.62) among 68 subjects during three months. The self-reported cumulative use agreed quite well with the operator records (3.2 versus 3.1 hours). In another German study [7], information on the duration of calls was obtained from software-modified phones for 45 subjects. The self-reported cumulative duration of calls showed a moderate correlation with the recorded time (r = 0.48), with some overestimation (mean call times 61.6 versus 53.8 minutes). In a large international study with 672 subjects from 11 countries [10], self-reported mobile phone use was evaluated in relation to operator records and software-modified phones. The overall correlation between the reported and recorded use was 0.69, both in terms of the number and duration of calls (kappa values 0.5 and 0.49, respectively). The duration of calls was overestimated by a factor of 1.42, while the number of calls was slightly underestimated. Retrospective exposure assessment based on interviews or questionnaires has major inherent uncertainties and constitutes a major source of error in case-control studies of mobile phone use.

This study confirms earlier findings that people tend to overestimate their mobile phone use [7-10,25,26]. However, some overestimation may be due to the formulation of the questions. The number and duration of calls were reported as ranges and cumulative exposure was estimated from the mid-points of the two intervals. This adds uncertainty, as the distribution within the reported range may be skewed, with the highest frequencies and durations occurring only rarely. However, this problem is difficult to avoid, as mobile phone use typically varies from day to day. Our findings also rely on the assumption of stability in mobile phone use over time, as the actual use records were obtained for a three-month period at the beginning of the year, which was not always the period for which the study subjects estimated their mobile phone use at recruitment (see the lower part of Figure 1). The sensitivity analysis restricted to the subjects with the best match for the periods of self-reported and operator-derived data showed only a slightly smaller mean difference between the two data sources when compared to the difference for the entire data set. This suggests that the discrepancy in the time period explains only a minor proportion of the observed differences between self-reported use and operator records. In the full study, the first three-month period of mobile phone data that is collected should be matched to the subjects' date of recruitment.

The full cohort study will collect exposure information both prospectively and retrospectively. Since historical operator records (typically more than 12 months) are not available, retrospective exposure information must be based on self-reported use only. However, the exposure assessment between self-reported and operator-derived data can be validated for this study. Retrospective exposure assessment can thus be based on self-reported mobile phone use corrected for subject-specific factors and will therefore probably be more accurate than unadjusted self-reported estimates.

As mobile phone use is not constant over time, the call duration during the six-month period before recruitment may not be representative of mobile phone use during the follow-up phase. For the full study, however, call data will be obtained for a three-month period each year. Therefore, misclassification due to seasonal variation is likely to be the major concern. This problem could be alleviated to some extent by acquiring anonymous or summary data on mobile phone use from the operators for the entire year and using it to validate the prediction based on three-month data.

The assessment of mobile phone use through operator databases is crucial, but these data were not received for 17% of the study participants, for whom prospective and retrospective exposure assessment could only be based on self-reported data. Operator data were probably missing due to a change of network operator or errors in the mobile phone numbers. Some subjects may have changed their network operator between the time the samples were gathered and the recruitment letters were sent. Operator data were unavailable from one of the three major network operators. Mobile traffic data were identified and obtained based on the mobile phone numbers only. Study participants gave their phone numbers both with the informed consent and in the questionnaire, and these numbers were entered into the database manually without double entry. Possible errors were found by comparing the numbers between these two sources and by checking the logic of the numbers (the length, the possible digits). However, study subjects often indicated their numbers on one form only or gave different numbers in different sources. In the full cohort study, there should be double entry for mobile phone numbers. By restricting the study to subjects with a maximum of two mobile phone numbers in use, estimation of the annual mobile phone use could be more accurate than in this pilot study.

Operator data may not completely represent the personal use of mobile phones. For example, study participants may use other people's phones (or vice versa), they may forget their phone numbers thus preventing operator data from being retrieved, or operator data may be wrongly linked. The questionnaire tried to address the problem of using other people's phones with the following alternatives for each phone: "Less than half of my calls have been made with this phone" and "More than half of my calls have been made with this phone." In the full study, the range of alternatives will be wider. The problem of forgetting mobile phone numbers is likely to be less relevant for prospective data collection, as mobile phone numbers are checked in the repeated questionnaires.

The study covered only private subscribers because the actual user cannot always be identified for corporate subscriptions. Neither can a private person give permission for a corporate subscription. This excludes a group of potentially heavy users with company mobile phones and may give rise to exposure misclassification if only their private secondary subscription data are obtained. However, the study questionnaire elicits information separately for each mobile phone. In this way, exposure due to company phones can be taken into account as self-reported data.

The radiofrequency exposure of mobile phones can be estimated from the exposure time and intensity of exposure. The exposure time can be calculated from operator-derived data adjusted for the mode of use (excluding use with hands-free devices). Studies of the determinants of power output (as a proxy for field strength) have given inconsistent results. In Sweden, substantial urban-rural differences were shown [28,29], while in Italy larger differences were observed between outdoor and indoor use [30], and regional differences dominated in the United States, [30-32]. In a German study, no strong and systematic determinants of output power were identified [7]. These factors will be covered in the study questionnaire, but their use in exposure assessment is challenging. It appears that the output power determinants depend on the network characteristics (design features and technical characteristics), and are therefore non-uniform in various settings. Furthermore, mobile phone use changes with new technologies and the study questionnaire should be revised and repeated regularly with new modes of use. If determination of the intensity of exposure could be improved with operator-derived data on output power and the network used (GSM versus UMTS), the exposure assessment would be more valid.

Ensuring a balanced distribution of the subjects with respect to age and sex was complicated by the fact that the owner of the subscription is not always the actual phone user (who is the target person and potential study participant). Operator records only include the subscription owner, but as minors are not allowed to open subscriptions, their phones are typically listed with the names of their parents. Since the study population was successfully established with a similar age and sex distribution across exposure strata, this is unlikely to be a problem in a prospective cohort study either. Moreover, if we have some overlap in demographic characteristics across exposure groups, incomplete balance can be adjusted for in the analyses.

Some 20% of the study subjects used at least two mobile phone numbers, usually with different network operators. Due to competition for market shares, operators use aggressive marketing tactics to attract customers and people change their network operators quite often. The regulation guaranteeing the possibility to maintain their old phone numbers has further increased switching between operators. The current network operator of each mobile phone number can be retrieved from a service provider maintained by the Finnish network operators. Thus, collaboration with all the major network operators is crucial to ensure comprehensive coverage of exposure. Information on changes in mobile phone numbers can be acquired from repeated questionnaires.

Based on the pilot study, the major challenges for a cohort study will be maximizing the participation rate in order to control the costs. The timing of the recruitment and ensuring media attention are likely to be important. The collection of information through web-based questionnaires may provide a possibility to increase participation. The Finnish regulation of research ethics does not allow the use of financial incentives. Other methods shown to increase response rates include personalized letters and questionnaires, colored ink, stamped return envelopes, reminders and questionnaires sent from universities rather than commercial organizations [33,34].

A prospective cohort study of mobile phone users and health appears to be feasible in Finland based on the pilot study. The recruitment of subjects from operators' databases was successful with a balanced distribution of exposures achieved in relation to age and sex. Furthermore, the retrieval of data on mobile phone use from operators' records was successful for the first year for both operators and all study years for one operator. The response rate was relatively low, but as an eventual full study would be based on internal comparisons, the low participation would affect the cost, but not the validity of the results. The low response rate may reflect the fact that Finns do not perceive a hazard from mobile phones or that mobile phones are such an integral part of everyday life that even a small risk would be acceptable (a situation comparable, for instance, to traffic-related health risks). Alternatively, a long follow-up with health information collected from multiple sources may affect the willingness to join the study. Finally, providing access to call records may be considered a sensitive issue due to privacy reasons. As there were no differences in the response rates between the recruitment methods, the less expensive two-phase recruitment seems preferable.

## Conclusions

Modest to fair agreement was found between self-reported and operator-derived estimates of mobile phone use. Overestimation of mobile phone use was common. The level of mobile phone use increased over time in a similar way for all mobile phone users. A prospective cohort study would be feasible in Finland, although the potentially low participation rate would increase the resources required for recruitment. Collaboration with the major Finnish network operators during the follow-up would be required for a successful study.

## List of abbreviations

GSM: Global System for Mobile Communication; RF: radiofrequency; UMTS: Universal Mobile Telecommunication System

## Competing interests

The authors declare that they have no competing interests.

## Authors' contributions

SH coordinated the study from 2007 onwards, took care of data management and descriptive statistics, and drafted the manuscript. KT coordinated the study until the end of 2006 and commented on the manuscript. PK participated in the coordination and helped to draft the manuscript. AA participated in study design and coordination, and in the drafting and revision of the manuscript. All authors read and approved the final manuscript.

## References

[B1] Ahlbom A Feychting M Green A Kheifets L Savitz DA Swerdlow AJ ICNIRP (International Commission for Non-Ionizing Radiation Protection) Standing Committee on Epidemiology Epidemiologic evidence on mobile phones and tumor risk: a review Epidemiology 2009 510 639 652 10.1097/EDE.0b013e3181b0927d 19593153

[B2] Kan P Simonsen SE Lyon JL Kestle JR Cellular phone use and brain tumor: a meta-analysis J Neurooncol 2008 86 71 78 10.1007/s11060-007-9432-1 17619826

[B3] Lahkola A Tokola K Auvinen A Meta-analysis of mobile phone use and intracranial tumors Scand J Work Environ Health 2006 32 171 177 16804618 10.5271/sjweh.995

[B4] Auvinen A Hietanen M Luukkonen R Koskela RS Brain tumors and salivary gland cancers among cellular telephone users Epidemiology 2002 13 356 359 10.1097/00001648-200205000-00018 11964939

[B5] Dreyer NA Loughlin JE Rothman KJ Cause-specific mortality in cellular telephone users JAMA 1999 282 1814 1816 10.1001/jama.282.19.1814-a 10573269

[B6] Schüz J Jacobsen R Olsen JH Boice JD Jr McLaughlin JK Johansen C Cellular telephone use and cancer risk: update of a nationwide Danish cohort J Natl Cancer Inst 2006 98 1707 1713 17148772 10.1093/jnci/djj464

[B7] Berg G Schuz J Samkange-Zeeb F Blettner M Assessment of radiofrequency exposure from cellular telephone daily use in an epidemiological study: German Validation study of the international case-control study of cancers of the brain--INTERPHONE-Study J Expo Anal Environ Epidemiol 2005 15 217 224 10.1038/sj.jea.7500390 15266354

[B8] Parslow C Hepworth SJ McKinney PA Recall of past use of mobile phone handsets Radiat Prot Dosimetry 2006 106 223 240 10.1093/oxfordjournals.rpd.a006354 14690324

[B9] Tokola K Kurttio P Salminen T Auvinen A Reducing overestimation in reported mobile phone use associated with epidemiological studies Bioelectromagnetics 2008 29 559 563 10.1002/bem.20424 18521851

[B10] Vrijheid M Cardis E Armstrong BK Auvinen A Berg G Blaasaas KG Brown J Carroll M Chetrit A Christensen HC Deltour I Feychting M Giles GG Hepworth SJ Hours M Iavarone I Johansen C Klaeboe L Kurttio P Lagario S Lonn S McKinney PA Montestrucq L Parslow RC Richardson L Sadetzki S Salminen T Schuz J Tynes T Woordward A Validation of short term recall of mobile phone use for the Interphone study Occup Environ Med 2006 63 237 243 10.1136/oem.2004.019281 16556742 PMC2078087

[B11] Vrijheid M Richardson l Armstrong BK Auvinen A Berg G Carroll M Chetrit A Deltour I Feychting M Giles GG Hours M Iavarone I Lagario S Lönn S McBride M Parent ME Sadetzki S Salminen T Sanchez M Schlehofer B Schüz J Siemiatycki J Tynes T Woodward A Yamaguchi N Cardis E Quantifying the impact of selection bias caused by nonparticipation in a case-control study of mobile phone use Ann Epidemiol 2009 19 33 41 10.1016/j.annepidem.2008.10.006 19064187

[B12] Lahkola A Salminen T Auvinen A Selection bias due to differential participation in a case-control study of mobile phone use and brain tumors Ann Epidemiol 2005 15 321 325 10.1016/j.annepidem.2004.12.009 15840544

[B13] Schüz J Elliott P Auvinen A Kromhaut H Harbo Poulsen A Johansen C Olsen JH Hillert L Feychting M Fremling K Toladano M Heinävaara S Slottje P Vermeulen R Ahlbom A An international prospective cohort study of mobile phone users and health (Cosmos): Design considerations and enrolment Cancer Epidemiol 2011 35 1 37 43 20810339 10.1016/j.canep.2010.08.001

[B14] National Radiological Protection Board (NRPB) Report of an independent advisory group on non-ionising radiation. Health effects from radiofrequency electromagnetic fields 2003 Chilton, Didcot: National Radiological Protection Board No 2

[B15] National Research Council of the National Academies Identification of Research Needs Relating to Potential Biological or Adverse Health Effects of Wireless Communication 2008 Washington D.C: The National Academies Press

[B16] Goldstein LS Dewhirst MW Repacholi M Kheifets L Summary, conclusions and recommendations: adverse temperature levels in the human body Int J Hyperthermia 2003 19 373 384 10.1080/0265673031000090701 12745976

[B17] European Commission Health Effects of Exposure to EMF (SCENIHR) 2009 European Commission http://ec.europa.eu/health/ph_risk/committees/04_scenihr/docs/scenihr_o_022.pdf

[B18] Abdel-Rassoul G El-Fateh OA Salem MA Michael A Farahat F El-Batanouny M Salem E Neurobehavioral effects among inhabitants around mobile phone base stations Neurotoxicology 2007 28 434 440 10.1016/j.neuro.2006.07.012 16962663

[B19] Chia SE Chia HP Tan JS Prevalence of headache among handheld cellular telephone users in Singapore: a community study Environ Health Perspect 2000 108 1059 1062 10.1289/ehp.001081059 11102297 PMC1240163

[B20] Huss A Röösli M Consultations in primary care for symptoms attributed to electromagnetic fields--a survey among general practitioners BMC Public Health 2006 6 267 10.1186/1471-2458-6-267 17074080 PMC1635563

[B21] Hutter HP Moshammer H Wallner P Kundi M Subjective symptoms, sleeping problems, and cognitive performance in subjects living near mobile phone base stations Occup Environ Med 2006 63 307 313 10.1136/oem.2005.020784 16621850 PMC2092490

[B22] Khan MM Adverse effects of excessive mobile phone use Int J Occup Med Environ Health 2008 21 289 293 10.2478/v10001-008-0028-6 19228576

[B23] Röösli M Radiofrequency electromagnetic field exposure and non-specific symptoms of ill health: a systematic review Environ Res 2008 107 277 287 18359015 10.1016/j.envres.2008.02.003

[B24] Sandström M Wilen J Oftedal G Hansson MK Mobile phone use and subjective symptoms. Comparison of symptoms experienced by users of analogue and digital mobile phones Occup Med 2001 51 25 35 10.1093/occmed/51.1.25 11235824

[B25] Funch D Rothman KJ Loughlin JE Dreyer NA Utility of telephone company records for epidemiologic studies of cellular telephones Epidemiology 1996 7 299 302 10.1097/00001648-199605000-00014 8728445

[B26] Samkange-Zeeb F Berg G Blettner M Validation of self-reported cellular phone use J Expo Anal Environ Epidemiology 2004 14 245 248 10.1038/sj.jea.7500321 15141153

[B27] Vrijheid M Deltour I Krewski D Sanchez M Cardis I The effects of recall errors and of selection bias in epidemiologic studies of mobile phone use and cancer risk J Expo Anal Environ Epidemiology 2006 16 371 384 10.1038/sj.jes.7500509 16773122

[B28] Hillert L Ahlbom A Neasham D Feychting M Jarup L Navin R Elliott P Call-related factors influencing output power from mobile phones J Expo Sci Environ Epidemiol 2006 16 507 514 10.1038/sj.jes.7500485 16670713

[B29] Lönn S Forssen U Vecchia P Ahlbom A Feychting M Output power levels from mobile phones in different geographical areas; implications for exposure assessment Occup Environ Med 2004 61 769 772 15317918 10.1136/oem.2003.012567 PMC1763677

[B30] Ardoino L Barbieri E Vecchia P Determinants of exposure to electromagnetic fields from mobile phones Radiat Prot Dosimetry 2004 111 403 406 10.1093/rpd/nch062 15550710

[B31] Erdreich LS Van Kerkhove MD Scrafford CG Barraj L McNeely M Shum M Sheppard AR Kelsh M Factors that influence the radiofrequency power output of GSM mobile phones Radiat Res 2007 168 253 261 10.1667/RR0906.1 17638408

[B32] Morrissey J Radiofrequency exposure in mobile phone users: Implications for exposure assessment in epidemiological studies Radiat Prot Dosimetry 2007 123 490 497 10.1093/rpd/ncl547 17213224

[B33] Edwards P Roberts I Clarke M Diguiseppi C Pratap S Wentz R Kwan I Increasing response rates to postal questionnaires: systematic review BMJ 2002 324 1183 10.1136/bmj.324.7347.1183 12016181 PMC111107

[B34] Edwards P Roberts I Clarke M Diguiseppi C Pratap S Wentz R Kwan I Cooper R Methods to increase response rates to postal questionnaires Cochrane Database Syst Rev 2007 MR000008 17443629 10.1002/14651858.MR000008.pub3

